# The seminal plasma sTLR4/IL-1β ratio as an independent factor associated with sperm chromatin condensation defects

**DOI:** 10.3389/fphys.2026.1917944

**Published:** 2026-08-21

**Authors:** Ying Zhao, Zegeng Lin, Liangju Chen, Fangmei Xie, Lin Zhong, Wenfeng Luo, Jinhua He, Mingyi Ye, Zeping Han

**Affiliations:** 1 Central Laboratory, The Affiliated Panyu Central Hospital, Guangzhou Medical University, Guangzhou, Guangdong, China; 2 Department of Laboratory Medicine, The Affiliated Panyu Central Hospital of Guangzhou Medical University, Guangzhou, Guangdong, China; 3 School of Life Science, South China Normal University, Guangzhou, China; 4 Department of Anesthesiology, The Affiliated Panyu Central Hospital of Guangzhou Medical University, Guangzhou, Guangdong, China; 5 Pediatrics Department,The Affiliated Panyu Central Hospital, Guangzhou Medical University, Guangzhou, Guangdong, China

**Keywords:** firth penalized regression, inflammatory-oxidative stress, seminal plasma, sperm chromatin condensation deficiency, sTLR4/IL-1β ratio

## Abstract

**Objective:**

To investigate the associations between seminal plasma inflammatory biomarkers, particularly the sTLR4/IL-1β ratio, and sperm chromatin condensation deficiency (SCCD) in men undergoing fertility evaluation, and to assess its potential utility in identifying individuals at risk for SCCD.

**Methods:**

74 men undergoing fertility evaluation were enrolled and categorized into a Normal SCCD group (SCCD ≤ 30%, n = 37) and an Impaired SCCD group (SCCD > 30%, n = 37) based on aniline blue staining. Seminal plasma levels of inflammatory markers, including HMGB1, sTLR4, IL-1β, TNF-α and oxidative stress markers, including 8-iso-PGF2α, total antioxidant capacity (T-AOC) were measured. Spearman correlation analysis, Firth’s penalized logistic regression, and receiver operating characteristic (ROC) curve analysis was employed to evaluate the discriminative performance of the biomarkers and the combined score, respectively.

**Results:**

Univariate Firth regression analysis revealed that the sTLR4/IL-1β ratio was a factor associated with reduced risk against SCCD (OR approaching 0, 95% CI: 0.00 - 0.04, *P* = 0.006), while T-AOC was significantly associated with increased risk of SCCD (OR = 200.99, 95% CI: 1.52 - 38205.02, *P* = 0.033). Multivariable analysis confirmed that sTLR4/IL-1β remained an independent factor associated with reduced SCCD risk after adjusting for confounders (*P* < 0.001), whereas T-AOC was not statistically significant in the multivariable model (*P* = 0.472). ROC curve analysis yielded an area under the curve (AUC) of 0.786 for sTLR4/IL-1β, indicating moderate-to-good discriminative ability.

**Conclusion:**

A higher seminal plasma sTLR4/IL-1β ratio is independently associated with reduced SCCD risk, suggesting that preserved immunological buffering may protect sperm chromatin integrity. The sTLR4/IL-1β ratio demonstrates moderate-to-good discriminatory ability (AUC = 0.786) and, as an exploratory finding, may serve as a potential auxiliary assessment tool in future larger validation studies.

## Introduction

Sperm DNA integrity is a critical determinant of male fertility potential, as its impairment is closely associated with fertilization failure, poor embryonic development, and miscarriage (Liu et al., 2024). Among the various pathological mechanisms leading to sperm DNA damage, oxidative stress (OS) and chronic inflammation are considered two core drivers. Evidence indicates that excessive reactive oxygen species (ROS) in seminal plasma can directly attack sperm DNA, causing base oxidation and strand breaks. Concurrently, microenvironmental imbalances mediated by inflammatory cytokines, such as IL-1β, TNF-α, further amplify this damage (Naseer et al., 2024).

Although conventional semen analysis remains the clinical cornerstone, it has limitations in assessing functional sperm quality, as routine parameters reflect mainly quantitative and gross morphological traits rather than nuclear maturation status (Gao et al., 2021). Parameters such as sperm DNA fragmentation (SDF) can reflect the extent of DNA damage but often lag behind early molecular-level imbalances, since chromatin condensation defects represent an earlier stage of nuclear immaturity than overt strand breaks (Oumaima et al., 2018). Previous studies have shown that SCCD, as defined by aniline blue positivity, exhibit inconsistent associations with conventional semen parameters. Hammadeh et al. (2001) reported no significant correlation between aniline blue positivity and sperm concentration, motility, or morphology, whereas Sellami et al. (2013) found that men with high chromatin immaturity showed no differences in sperm concentration, total motility, or percentage of normal forms compared with those with low immaturity, yet exhibited significantly higher rates of head abnormalities and microcephalic spermatozoa. These findings indicate that a subset of men with normal routine parameters may still harbor significant chromatin immaturity, and that conventional semen analysis alone cannot reliably exclude SCCD. Therefore, investigating earlier biomarkers of inflammation and oxidative stress in seminal plasma is of great value for predicting and intervening in the risk of SCCD, particularly in individuals with ostensibly normal routine semen parameters but underlying chromatin immaturity. In recent years, high mobility group box 1 (HMGB1) in seminal plasma has garnered attention as a novel damage-associated molecular pattern (DAMP), released by damaged or stressed cells to initiate immune responses. Soluble Toll-like receptor 4 (sTLR4), in contrast, functions as a decoy receptor that recognizes and neutralizes DAMPs such as HMGB1, thereby limiting the activation of the TLR4/NF-κB signaling cascade (Zhang et al., 2015; Chu et al., 2018). Together, the balance between HMGB1 and sTLR4 reflects the net inflammatory status of the seminal microenvironment: excessive HMGB1 relative to sTLR4 may lead to uncontrolled immune activation, while adequate sTLR4 buffering may protect against inflammatory damage. However, existing studies often examine individual factors in isolation, overlooking this dynamic balance between activating and inhibitory components within the inflammatory pathway. Disruption of this balance may more accurately reflect the pathophysiology of SCCD than the absolute value of any single marker (Gupta et al., 2021).

**Figure 1:**
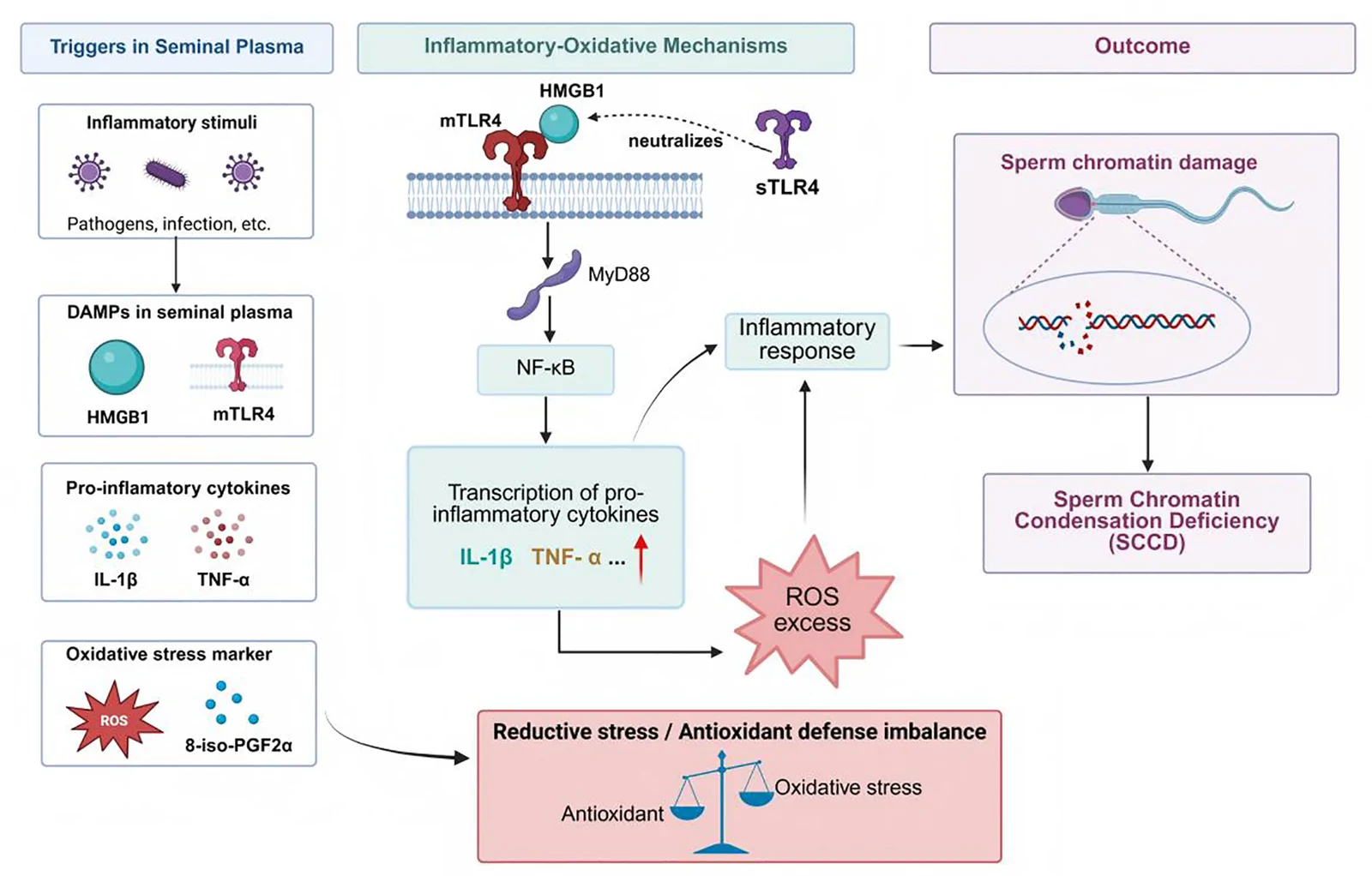
Schematic overview of the proposed mechanisms linking seminal plasma inflammation and oxidative stress to SCCD. Figure created with BioRender.com.

**Figure 1** illustrates the established signaling pathways linking seminal plasma inflammatory and oxidative stress triggers to SCCD. In the seminal microenvironment, DAMPs such as HMGB1 can bind to membrane-bound Toll-like receptor 4 (mTLR4) on sperm or local immune cells (Chu et al., 2018; Cao et al., 2025). This ligation activates the MyD88-dependent NF-κB signaling cascade, leading to the transcription of pro-inflammatory cytokines and the generation of ROS (Zhang et al., 2015; Chu et al., 2018). sTLR4 acts as a decoy receptor, neutralizing HMGB1 and limiting this cascade (Chu et al., 2018), while total antioxidant capacity (T-AOC) counterbalances ROS (Gupta et al., 2021). Disruption of this homeostatic balance, characterized by an inadequate sTLR4-mediated immune buffer and an insufficient antioxidant defense relative to oxidative pressure, can result in SCCD (Zhang et al., 2015; Cao et al., 2025). This conceptual framework underpins the hypotheses tested in the present study.

Building upon this background, the present study aimed to systematically evaluate the associations between SCCD risk and seminal plasma biomarkers related to inflammation and oxidative stress. To achieve this, we innovatively constructed ratio indices, such as sTLR4/IL-1β and sTLR4/HMGB1, to quantify the net activation level of inflammatory pathways and mitigate multicollinearity. Subsequently, Firth’s penalized maximum likelihood regression was employed to correct for potential small-sample bias. The present study was therefore exploratory in nature, aiming to generate hypotheses regarding the role of seminal inflammatory biomarkers in SCCD rather than to confirm predefined associations.

## Materials and methods

### Study participants

This comparative cross-sectional study consecutively recruited men of reproductive age who attended fertility evaluation at the Affiliated Panyu Central Hospital, Guangzhou Medical University, between January 2025 and December 2025. Semen samples were collected and processed according to the criteria recommended by the World Health Organization (WHO) Laboratory Manual for the Examination and Processing of Human Semen, 6th edition (World Health Organization, 2021).

The classification of SCCD was based on aniline blue staining. The 30% cutoff was adopted according to the manufacturer’s instruction of the aniline blue staining kit (BRED Life Science Technology Inc., Shenzhen, China), where ≤ 30% positive staining indicates normal chromatin maturation and > 30% indicates condensation deficiency. This threshold is consistent with prior reports using a 30% cutoff (World Health Organization, 2021), with the only difference being the assignment of the exact 30% value (≤ 30% vs <no><</no> 30% as normal), which does not affect the substantive classification.

Participants were stratified according to the SCCD rate, determined by aniline blue staining:

1. Normal SCCD group: SCCD ≤ 30%
2. Impaired SCCD group: SCCD > 30%

Thirty-seven participants were allocated to each group.

### Inclusion criteria

1. Abstinence period of 2–7 days prior to semen collection;
2. Normal semen liquefaction time (≤ 60 minutes at 37 °C);

### Exclusion criteria

1. Genital developmental abnormalities, clinically significant varicocele, or a history of related surgery;
2. Leukocytospermia: Leukocytospermia was defined as ≥1.0 × 10^6^ peroxidase-positive white blood cells per milliliter, as determined by the peroxidase staining method (BRED Life Science Technology Inc., Shenzhen, China, catalog number: 20250801), according to the WHO laboratory manual (World Health Organization, 2021);
3. Complaints of sexual dysfunction;
4. Presence of chronic systemic diseases or known genetic disorders potentially affecting fertility;
5. Current or recent (within 3 months) urogenital tract infection.

The study protocol was approved by the Ethics Committee of Panyu Central Hospital, Guangzhou Medical University (Approval No.: PYRC-2026-171-01) and was conducted in accordance with the principles of the Declaration of Helsinki.

### Semen sample collection and analysis

Participants were instructed to observe a sexual abstinence period of 2 to 7 days prior to sample collection. Complete ejaculates were obtained by masturbation into sterile, wide-mouthed, non-toxic specimen cups. After liquefaction at 37 °C, samples were delivered to the laboratory for analysis within 1 hour of collection. Liquefied semen samples were analyzed using a computer-assisted semen analysis system to assess the following parameters: semen volume, sperm concentration, total sperm motility, progressive motility, and rapid progressive motility. Sperm morphology was evaluated using Diff-Quik staining (staining solution purchased from Micropticsl, Spain) in accordance with the criteria outlined in the World Health Organization Laboratory Manual for the Examination and Processing of Human Semen(6th edition) (World Health Organization, 2021).

Sperm chromatin condensation was assessed using aniline blue staining, which specifically binds to residual histones in incompletely matured sperm nuclei, indicating defective histone-to-protamine replacement. The aniline blue staining kit was purchased from BRED Life Science Technology Inc. (Shenzhen, China). Briefly, semen samples were washed twice with phosphate-buffered saline (PBS) and smears were prepared on clean glass slides. After air-drying, the smears were fixed in 3% glutaraldehyde in PBS for 30 minutes at room temperature. The slides were then stained with 5% aniline blue (dissolved in 4% acetic acid) for 10 minutes, followed by rinsing with distilled water and dehydration through a graded ethanol series. A minimum of 200 spermatozoa per sample were evaluated under bright-field microscopy (X-Ultra, Shanghai LeiTe Technology Co., Ltd, China) at 1000× magnification.

To obtain seminal plasma for inflammatory marker assessment, freshly liquefied semen samples were centrifuged at 3,000 rpm for 15 minutes at room temperature. The seminal plasma was carefully aspirated, aliquoted, and immediately stored at -80 °C until assay to prevent freeze-thaw cycles. The concentrations of inflammatory and oxidative stress biomarkers, including IL-1β, TNF-α, HMGB1, sTLR4, 8-iso-PGF2α and T-AOC, in seminal plasma were quantified using commercially available enzyme-linked immunosorbent assay kits. All procedures were strictly performed in accordance with the manufacturer’s instructions (Guangzhou Manrui Biotechnology Co., Ltd., Guangzhou, China). All samples were run in duplicate, and a standard curve with known concentrations was included in each assay to ensure accuracy and reproducibility.

### Statistical analysis

Statistical analyses were performed using GraphPad Prism 11 (GraphPad Software, San Diego, CA, USA). The normality of continuous variables was assessed using the Shapiro-Wilk test. Continuous variables that followed a normal distribution are presented as the mean ± standard deviation (SD) and were compared between groups using the independent samples t-test. Variables that were not normally distributed are presented as the median with interquartile range [M (Q1, Q3)] and were compared using the Mann-Whitney U test. All tests were two-sided, and a P-value of less than 0.05 was considered statistically significant.

To identify biomarkers associated with SCCD and assess their discriminative performance, the following analyses were conducted using R software (version 4.4.2). First, correlations among inflammatory and oxidative stress markers were examined using Spearman’s rank correlation analysis. Subsequently, univariate and multivariable Firth’s penalized maximum likelihood logistic regression models were fitted to obtain robust parameter estimates and mitigate potential bias from small sample sizes or data separation.

The discriminatory performance of the classification models was evaluated using receiver operating characteristic (ROC) curves and the area under the curve (AUC). Specifically, ROC curves were generated for individual significant biomarkers and for a novel combined score, which was derived by standardizing and summing the key variables.

## Results

### Demographic characteristics

Characteristics of the study participants, including mean age, basic sperm parameters, and SCCD percentage details are shown in **Table 1** were compared. There were no significant differences in age, abstinence time, semen volume, sperm concentration, total motility, progressive motility, rapid progressive motility, or normal morphology between the Impaired SCCD group and the Normal SCCD group (all *P* > 0.05). However, the percentage of SCCD was significantly higher in the Impaired SCCD group compared to the Normal SCCD group (median [IQR]: 46.00% [36.00–58.00] vs 16.00% [10.00–22.00], *P* < 0.0001, Mann-Whitney U test). Additionally, semen pH was slightly higher in the Impaired SCCD group (median [IQR]: 7.50 [7.40, 7.50]) than in the Normal SCCD group (7.40 [7.30, 7.50]), with a marginal significance (*P* = 0.0126).

**Table 1:** Comparison of demographic, semen, and clinical characteristics between the normal and impaired SCCD groups.

Characteristics	Impaired SCCD group (n=37)	Normal SCCD group (n=37)	*Mann-Whitney* U/t-test	*P* value
Age (years)	33.08 ± 4.61	33.57 ± 5.86	0.397	0.6927
Abstinence time (days)	4.00 (3.00, 6.00)	5.00 (3.50, 6.00)	641	0.6354
Semen volume (mL)	4.59 ± 1.31	4.77 ± 1.35	0.587	0.5591
Semen pH	7.50 (7.40, 7.50)	7.40 (7.30, 7.50)	470	0.0126
Sperm Concentration (10^6^/mL)	35.70 (29.65, 85.50)	54.60 (23.90, 97.90)	604.5	0.3908
Total Motility (A+B+C, %)	38.90 ± 13.71	41.37 ± 14.95	0.742	0.4602
Progressive Motility (A+B, %)	32.28 ± 13.85	35.31 ± 14.16	0.931	0.3552
Rapid Progressive Motility (A, %)	23.46 ± 10.99	27.33 ± 12.98	1.384	0.1706
Normal Morphology (%)	4.65 (2.45, 6.20)	4.80 (2.05, 6.30)	649	0.8542
SCCD (%)	46.00 (36.00, 58.00)	16.0 (10.00, 22.00)	0	< 0.0001

Data are presented as mean ± standard deviation (SD) for normally distributed variables or median (interquartile range, IQR) for non-normally distributed variables. Independent samples t-test (for normally distributed variables) or Mann-Whitney U test (for non-normally distributed variables) was used to compare groups.

### Sperm nuclear protein staining

**Figure 2:**
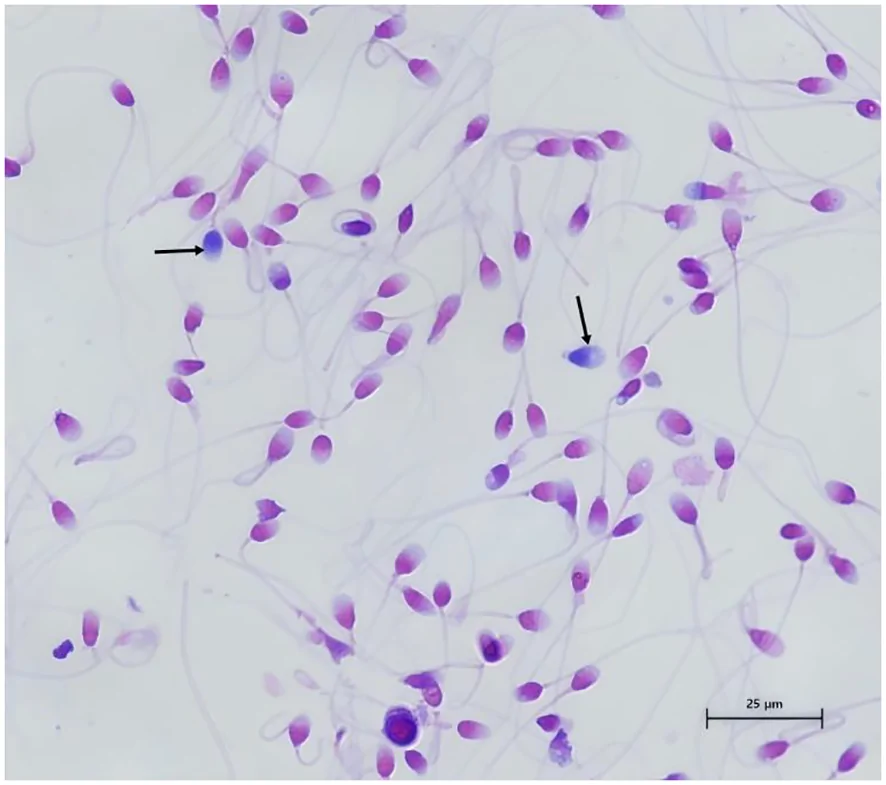
Representative photomicrographs of sperm nuclear protein staining by aniline blue.

Sperm samples were subjected to aniline blue staining to assess the distribution of nuclear proteins, as described in the Materials and Methods section. The staining results are shown in **Figure 2**. Under high-power microscopy (Shanghai LeiTe Technology Co. Ltd, X-Ultra; 100× objective lens), normal spermatozoa exhibited dense, magenta-purple staining, reflecting intact and well-condensed chromatin. In contrast, abnormal spermatozoa displaying nuclear protein deficiency: characteristic of SCCD, were observed with significantly lighter, pale-blue staining (highlighted by black arrows). Scale bar: 25 μm.

### Distinct seminal plasma profile: higher inflammation and lower sTLR4-mediated buffering in impaired SCCD

Regarding seminal plasma biomarkers, men with impaired sperm chromatin condensation displayed a distinct pro-inflammatory and oxidative profile. Levels of HMGB1, sTLR4, IL-1β, TNF-α, and 8-iso-PGF2α were all significantly higher in the Impaired SCCD Group than in the Normal SCCD Group (*P* < 0.05 for all). However, the T-AOC showed no significant difference between the two groups (*P* = 0.2243). To assess the homeostatic balance between the decoy receptor sTLR4 and inflammatory drivers, we calculated the sTLR4/HMGB1 and sTLR4/IL-1β ratios. Both ratios were significantly higher in the Normal SCCD group compared to the Impaired group (sTLR4/HMGB1: 0.122 ± 0.017 vs. 0.105 ± 0.013, *P* < 0.0001; sTLR4/IL-1β: 0.553 [IQR: 0.520–0.600] vs. 0.455 [IQR: 0.409–0.551], *P* < 0.0001). This indicates a superior capacity to buffer inflammatory signals in individuals with intact sperm chromatin condensation (**Table 2**).

**Table 2:** Comparison of seminal plasma inflammatory and oxidative stress biomarkers between the normal and impaired SCCD groups.

Parameters	Impaired SCCD group (n=37)	Normal SCCD group (n=37)	*Mann-Whitney* U/t-test	*P* value
HMGB1 (ug/L)	144.4 ± 14.02	136.0 ± 18.83	2.173	0.0331
sTLR4 (ng/mL)	15.11 ± 1.731	16.35 ± 1.256	3.552	0.0007
IL-1β (pg/mL)	31.53 (29.24, 35.28)	28.78 (27.42, 31.66)	404	0.0022
TNF-α (ng/L)	784.3 ± 49.72	750.7 ± 54.60	2.761	0.0073
8-iso-PGF2α (ng/L)	191.9 (160.3, 229.4)	218.8 (195.4, 289.8)	456	0.0131
T-AOC (μmol/mL)	0.158 (0.094, 0.254)	0.130 (0.071, 0.186)	571.5	0.2243
sTLR4/HMGB1	0.105 ± 0.013	0.122 ± 0.017	4.830	<0.0001
sTLR4/IL-1β	0.455 (0.409, 0.551)	0.553 (0.520, 0.600)	294	<0.0001

### Correlations between seminal plasma biomarkers, semen parameters, and SCCD

**Figure 3:**
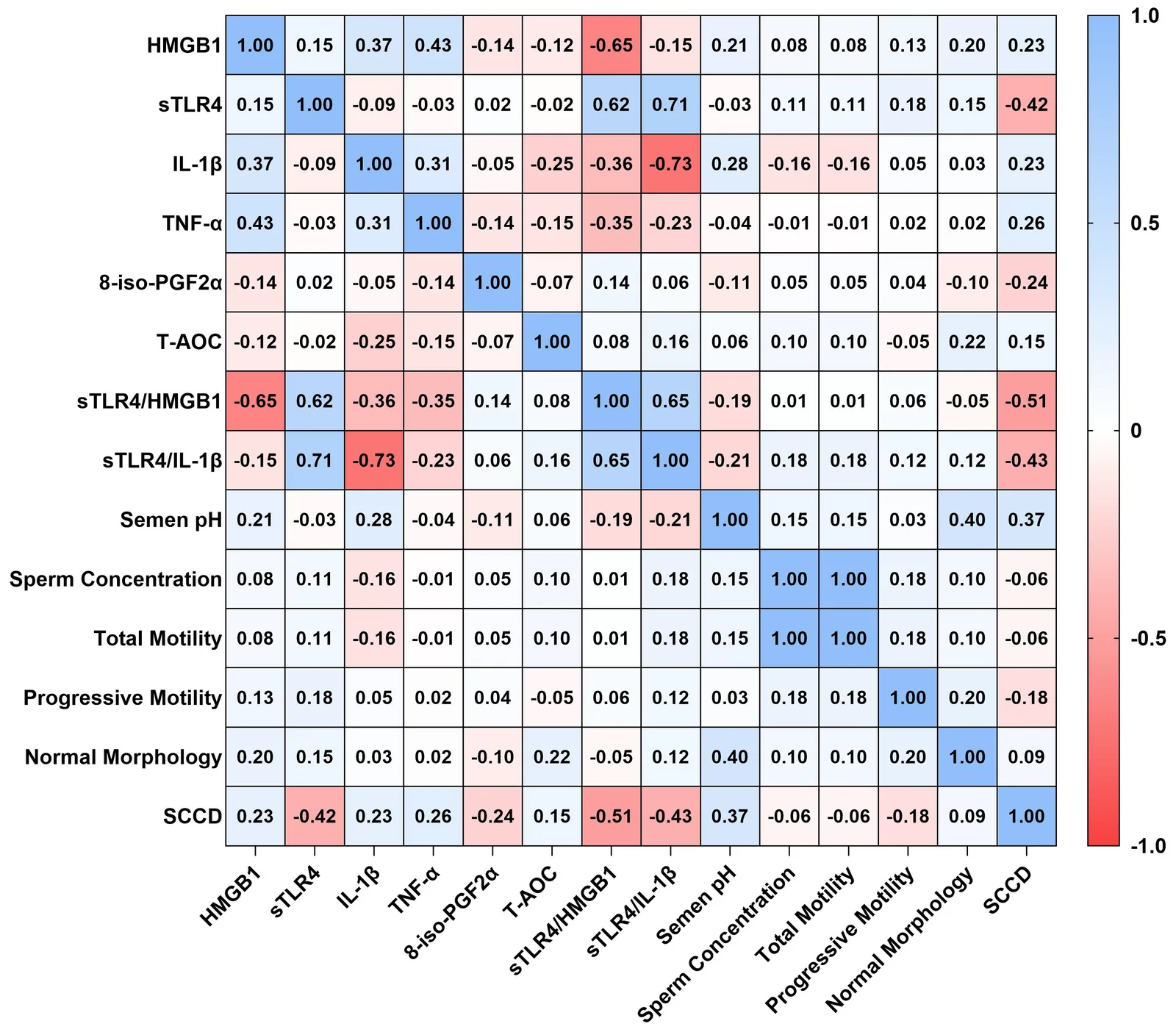
Spearman correlation matrix of seminal plasma inflammatory/oxidative stress biomarkers, semen parameters, and SCCD.

Correlation analysis revealed a complex network of associations among seminal plasma inflammatory and oxidative stress markers. Among the inflammatory markers themselves, HMGB1 showed significant positive correlations with both IL-1β (*r* = 0.37, *P* = 0.001) and TNF-α (*r* = 0.43, *P* < 0.001). A positive correlation was also observed between IL-1β and TNF-α (*r* = 0.31, *P* < 0.01), suggesting a potential synergistic role of these pro-inflammatory factors in the inflammatory response. Furthermore, IL-1β was negatively correlated with T-AOC (*r* = -0.25, *P* < 0.05). Moreover, both IL-1β and TNF-α were negatively correlated with sTLR4/HMGB1, with correlation coefficients of *r* = -0.36 (*P* < 0.05) and *r* = -0.35 (*P* < 0.01), respectively. Notably, links were also found between inflammatory markers and routine semen parameters. IL-1β was positively correlated with semen pH (*r* = 0.28, *P* < 0.05), indicating that inflammatory activity may be associated with alterations in the acidity of the seminal microenvironment. When examining the relationships between these markers and semen quality parameters, the associations of sTLR4, sTLR4/HMGB1 and sTLR4/IL-1β with SCCD were most prominent. The detailed correlation strengths and statistical data for these specific associations with SCCD are presented below and illustrated in **Figure 3**.

**Figure 4:**
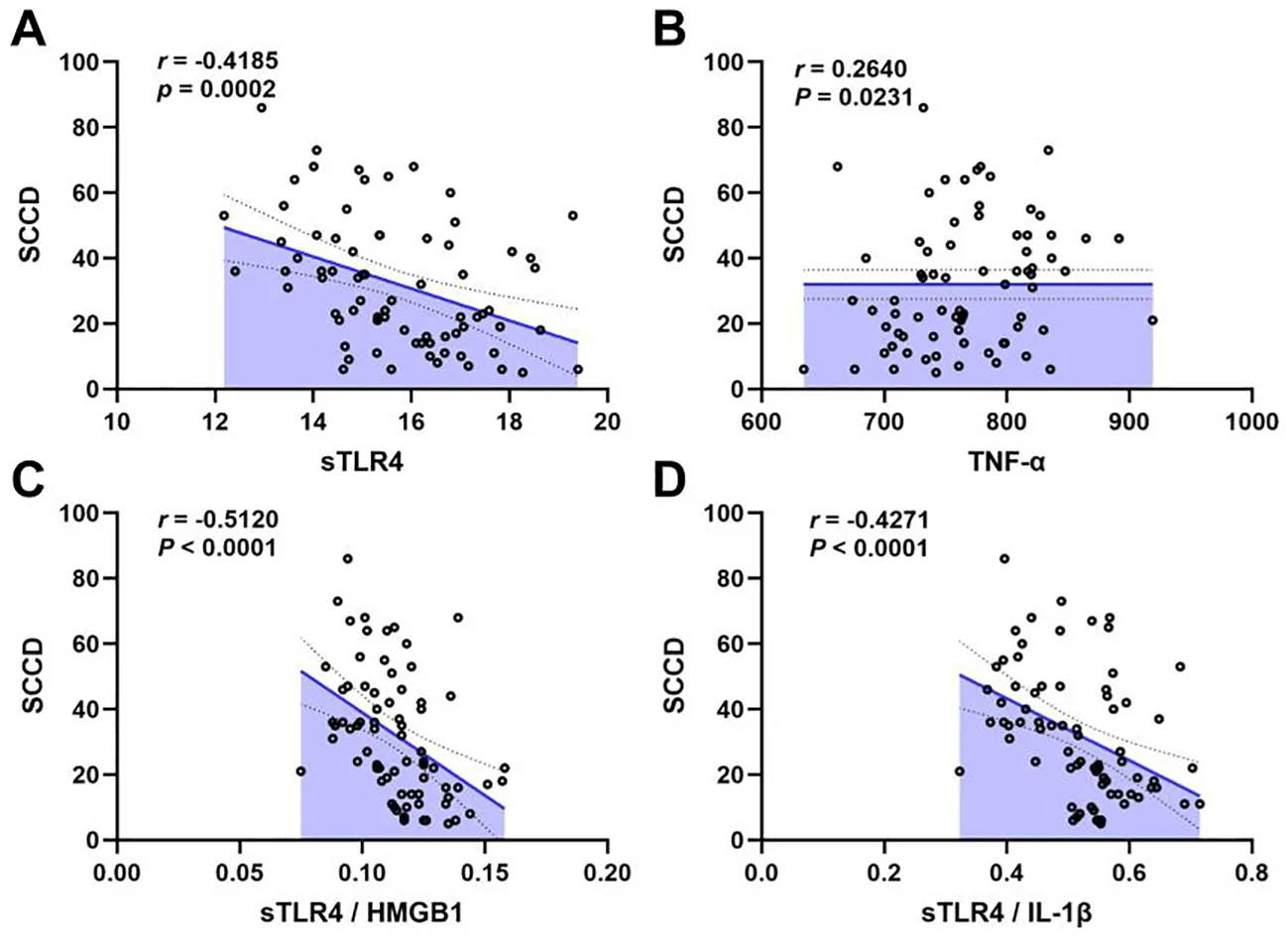
Scatter plots with linear regression fits showing Spearman correlations between seminal plasma biomarkers and SCCD rate. **(A)** sTLR4 vs. SCCD; **(B)** TNF-α vs. SCCD; **(C)** sTLR4/HMGB1 ratio vs. SCCD; **(D)** sTLR4/IL-1β ratio vs. SCCD. Correlation coefficients (r) and *P* values are shown in each plot. *Spearman’s* rank correlation coefficient (*r*) and *P*-value are shown. Solid lines represent linear regression fits, and dashed lines represent 95% confidence intervals.

As shown in **Figure 4**, Spearman’s correlation analysis provided a detailed quantitative assessment of the relationships between key seminal plasma markers and SCCD. A significant negative correlation was observed between sTLR4 and SCCD (*r* = −0.4185, *P* = 0.0002; **Figure 4A**). Notably, this inverse relationship was markedly strengthened when sTLR4 concentration was normalized to its pro-inflammatory ligands. The sTLR4/HMGB1 and sTLR4/IL-1βratios demonstrated robust negative correlations with SCCD, with correlation coefficients as high as −0.5120 (*P* < 0.0001; **Figure 4C**) and −0.4271 (*P* < 0.0001; **Figure 4D**), respectively. The sTLR4/HMGB1 ratio exhibited the strongest association with SCCD among all analyzed markers. In contrast, the pro-inflammatory cytokine TNF-α showed a significant positive correlation with SCCD (*r* = 0.2640, *P* = 0.0231; **Figure 4B**), suggesting its potential role in promoting abnormal chromatin structure. The oxidative stress marker 8-iso-PGF2α showed a weaker negative correlation with SCCD (*r* = −0.2353, *P* = 0.0436). HMGB1 was weakly positively correlated with SCCD (*r* = 0.2209, *P* = 0.0446), while the positive association between IL-1β and SCCD was of borderline statistical significance (*r* = 0.2282, *P* = 0.0506). No significant correlation was found between T-AOC and SCCD (*r* = 0.1549, *P* = 0.1876).

### Factors associated with sperm chromatin condensation deficiency

To investigate the association between seminal plasma inflammatory biomarkers and the risk of SCCD, we performed univariate Firth penalized-likelihood logistic regression to mitigate small-sample bias. As shown in **Table 3**, T-AOC was significantly associated with increased SCCD risk (adjusted OR = 200.99, 95% CI: 1.52 - 38205.02, *P* = 0.033), however, the extremely wide confidence interval suggests that this magnitude is highly unstable and should be interpreted with caution. The ratio sTLR4/IL-1β showed a statistically significant but imprecise association with reduced SCCD risk (adjusted OR ≈ 0.00, 95% CI: 0.00 - 0.04, *P* = 0.006), reflecting substantial estimation uncertainty despite the nominal significance. Neither TNF-α nor 8-iso-PGF2α demonstrated statistically significant associations with SCCD (*P* > 0.05). Although sTLR4/HMGB1 also suggested a directionally protective trend (adjusted OR ≈ 0.00), it did not reach statistical significance (*P* = 0.198), and its extraordinarily wide confidence interval (0.00 - 3171208.38) indicates that the association is inconclusive and likely unreliable due to model instability.

**Table 3:** Univariate firth penalized logistic regression analysis of biomarkers associated with SCCD risk.

Variables	OR	95% CI	*P*_value
TNF-α	1.01	1.00 ~ 1.02	0.223
8-iso-PGF2α	0.99	0.98 ~ 1.00	0.092
T-AOC	200.99	1.52~ 38205.02	0.033
sTLR4/HMGB1	0.00	0.00 ~ 3171208.38	0.198
sTLR4/IL-1β	0.00	0.00 ~ 0.04	0.006

### Multivariable logistic regression analysis

Variables that were statistically significant in univariate analysis (sTLR4/IL-1β and T-AOC), along with clinically relevant confounders including age, abstinence duration, semen pH, and total sperm motility, were entered into a multivariable Firth logistic regression model. After adjusting for these confounders, sTLR4/IL-1β remained significantly associated with SCCD (OR = 1.214×10^-6^, 95% CI: 3.427×10^-^¹^0^ - 9.998×10^-4^, *P* < 0.001), indicating that sTLR4/IL-1β is an independent factor associated with reduced risk of SCCD. In contrast, T-AOC was not statistically significant in the multivariable model (OR = 3.951, 95% CI: 0.095 - 214.308, *P* = 0.4716), suggesting that its association observed in univariate analysis may be confounded by other variables. The results of the multivariable analysis are presented in **Table 4**.

**Table 4:** Multivariable firth penalized logistic regression analysis of factors independently associated with SCCD risk.

Variable	Coefficient	SE	χ²	*P*-value	OR	95% CI
Model A: sTLR4/IL-1β + Confounders						
sTLR4/IL-1β	-1.362	2.663	18.565	< 0.001	1.214e-06	3.427e-10 ~ 9.998e-04
Age	-0.0001	0.048	1.110e-06	0.999	0.999	0.908 ~ 1.101
Abstinence Duration	0.138	0.179	0.586	0.444	1.148	0.806 ~ 1.653
Semen pH	-0.182	0.217	0.700	0.403	0.833	0.530 ~ 1.275
Total Motility	-0.006	0.018	0.096	0.757	0.994	0.959 ~ 1.031
Model B: T-AOC + Confounders						
T-AOC	1.374	1.920	0.518	0.4716	3.951	0.095 ~ 214.308
Age	-0.019	0.044	0.191	0.6618	0.981	0.897 ~ 1.070
Abstinence Duration	0.0012	0.159	5.394e-05	0.9941	1.001	0.730 ~ 1.374
Semen pH	-0.109	0.190	0.331	0.5651	0.897	0.611 ~ 1.300
Total Motility	-0.013	0.016	0.662	0.4158	0.987	0.955 ~1.019

### ROC curve analysis of sTLR4/IL-1β

**Figure 5:**
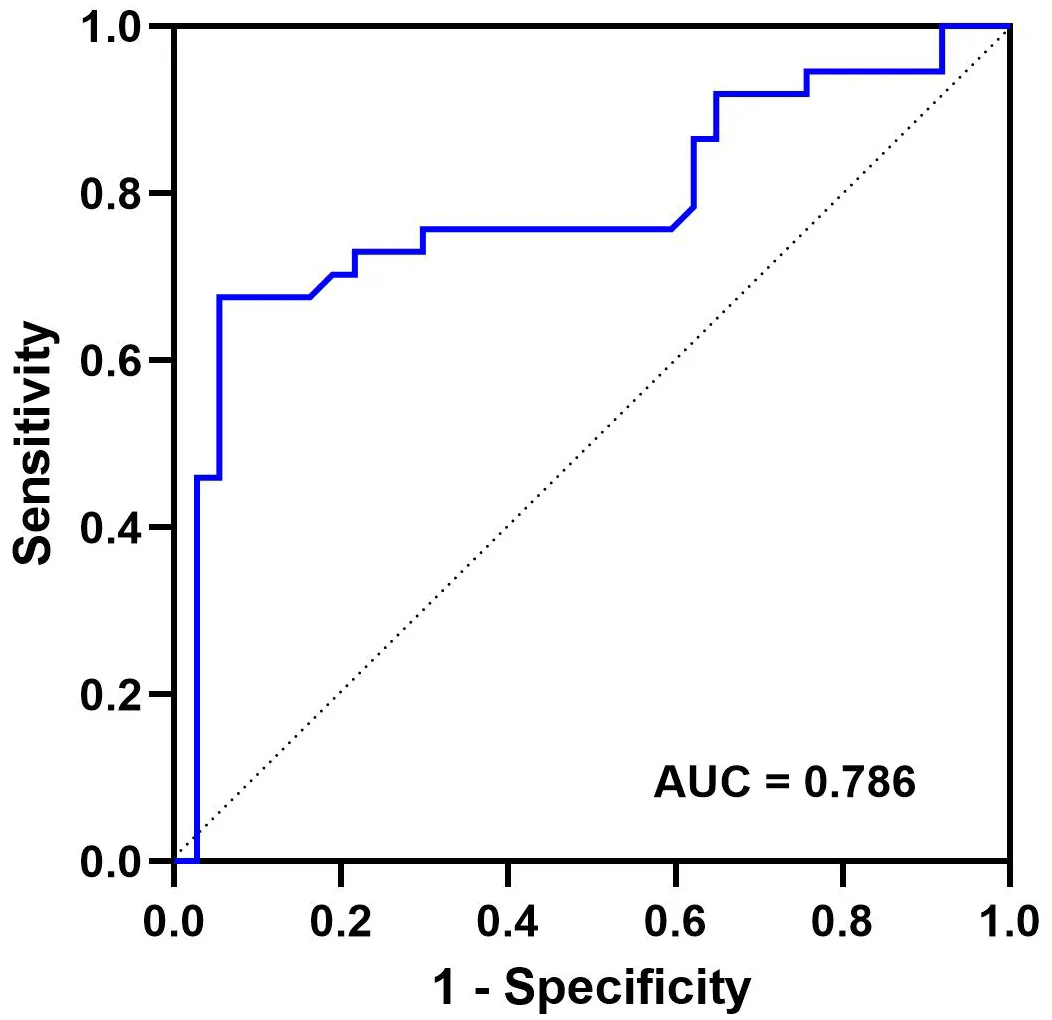
ROC curve of the sTLR4/IL-1β ratio for discriminating between the impaired SCCD and normal SCCD groups.

Receiver operating characteristic (ROC) curve analysis was performed to evaluate the discriminatory performance of sTLR4/IL-1β in distinguishing patients with SCCD from controls. The analysis yielded an area under the curve (AUC) of 0.786 (**Figure 5**), indicating moderate-to-good discriminative ability. This finding demonstrates the potential utility of sTLR4/IL-1β as a discriminatory biomarker for SCCD, consistent with the results of the multivariable Firth logistic regression analysis.

The gray dashed diagonal line represents the reference of no discrimination (AUC = 0.5). The AUC for sTLR4/IL-1β was 0.786.

## Discussion

The baseline characteristics revealed that the two groups were well-balanced in terms of age, abstinence time, and conventional semen parameters, except for semen pH. The Impaired SCCD group exhibited a marginally higher median semen pH compared to the Normal SCCD group. This finding aligns with previous observations that defective sperm chromatin condensation is associated with alterations in seminal plasma composition. Although the physiological mechanism is not fully elucidated, a more alkaline seminal environment might reflect a shift in the secretory function of accessory glands (World Health Organization, 2021), which could indirectly influence chromatin stability by modulating the redox balance (Zhou et al., 2026). However, given the relatively small absolute difference in pH values and the borderline significance, the clinical relevance of this finding warrants further investigation in larger cohorts.

Interestingly, no significant differences were observed in conventional semen parameters, such as sperm concentration, motility, and morphology, between the two SCCD groups. This finding may be attributed to several factors. First, both groups were drawn from the same source population of men attending fertility evaluation, rather than comparing fertile controls with infertile patients, which may reduce between-group variability in routine parameters. Second, previous studies have reported inconsistent associations between SCCD and conventional semen parameters (Hammadeh et al., 2001; Sellami et al., 2013), suggesting that chromatin condensation status does not always correlate with standard measures. Third, the relatively modest sample size may limit the statistical power to detect subtle differences. Notably, our correlation analysis revealed that IL-1β was positively correlated with semen pH (r = 0.28, *P* < 0.05), which is consistent with previous observations that genital tract inflammation is associated with elevated seminal pH (Garcia-Segura et al., 2020; Pérez-Soto et al., 2021). Although HMGB1 did not show direct correlations with individual semen parameters in our cohort, accumulating evidence indicates that HMGB1 acts as a key DAMP that binds TLR4 and activates the NF-κB cascade, thereby inducing downstream pro-inflammatory cytokines and ROS that can compromise sperm chromatin maturation (Cao et al., 2025). These findings highlight the clinical importance of assessing chromatin integrity and inflammatory markers in seminal plasma, even in men with normal routine semen parameters, reinforcing the notion that SCCD represents a dimension of sperm quality partially independent of conventional analysis.

Among these complementary biomarkers, T-AOC deserves particular attention, as it exhibited an intriguing dissociation from oxidative stress markers in our cohort. T-AOC is an integrative *in vitro* measure that aggregates radical-scavenging contributions from multiple seminal plasma constituents into a single value. As Fraga et al. emphasized, such assays estimate a global antioxidant potential but do not resolve which specific molecules are responsible, nor do they reflect the actual activity of key antioxidant enzymes such as superoxide dismutase or catalase (Fraga et al., 2014). Therefore, unchanged T-AOC does not exclude consumption of particular antioxidants or dysfunction of enzyme-based defenses. Oxidative stress is fundamentally defined as a dynamic imbalance where ROS generation exceeds antioxidant neutralization capacity, rather than a mere reduction in T-AOC (Jomova et al., 2023). Thus, elevated oxidative markers can occur even when T-AOC remains unchanged, provided that ROS production is sufficiently increased by inflammatory activation or mitochondrial dysfunction. Under chronic oxidative challenge, the antioxidant pool may be maintained or upregulated as a compensatory response. Petelin et al. observed significantly higher serum T-AOC in overweight adults, interpreting this as a compensatory response to ROS-driven oxidative stress (Petelin et al., 2017). In the present study, the unchanged seminal T-AOC in the impaired SCCD group, alongside elevated oxidative markers, may similarly represent a compensated antioxidant state rather than antioxidant deficiency. Taken together, the absence of a significant between-group difference in T-AOC (**Table 2**) and its nominally significant but highly unstable association in univariate regression (**Table 3**) are not contradictory. Rather, they reflect the fact that T-AOC is a composite measure influenced by compensatory dynamics and confounding covariates. This interpretation is further supported by the attenuation of the T-AOC effect after multivariable adjustment (**Table 4**), indicating that the univariate finding was likely confounded rather than representing an independent biological effect.

Unlike T-AOC, the sTLR4-related ratios exhibited a more consistent pattern across groups. Despite significantly elevated pro-inflammatory cytokines, such as HMGB1, IL-1β, TNF-α, in the impaired SCCD group, the normal SCCD group exhibited higher levels of sTLR4, sTLR4/HMGB1 and sTLR4/IL-1β, suggesting that sTLR4 may play a critical role in maintaining immunological homeostasis and protecting sperm chromatin integrity. As a soluble decoy receptor, sTLR4 can buffer inflammatory damage through multiple mechanisms. On one hand, the sTLR4/HMGB1 ratio directly reflects the capacity of sTLR4 to neutralize HMGB1, a core damage signal, at the source. By competitively binding HMGB1, sTLR4 prevents its engagement with membrane-bound TLR4 (mTLR4) and the subsequent activation of the NF-κB pathway, thereby providing protection at the initial stage of inflammation (Chu et al., 2018). In the male reproductive system, overactivation of the HMGB1/TLR4 axis has been shown to drive testicular inflammation and impair spermatogenesis (Cao et al., 2025). On the other hand, the sTLR4/IL-1β ratio may indicate the “network regulatory” role of sTLR4 within the inflammatory cascade. IL-1β is a key downstream product of NF-κB signaling and a potent amplifier of inflammation. A higher sTLR4/IL-1β ratio suggests that in individuals with intact sTLR4 function, the system may more effectively suppress the production or activity of secondary inflammatory mediators like IL-1β, thereby preventing runaway amplification of inflammatory signals (Dinarello, 2009). In our study, the higher sTLR4 levels and ratios in the normal group may effectively “buffer” the detrimental effects of a pro-inflammatory microenvironment, safeguarding the normal assembly and maturation of sperm nuclear proteins. Conversely, in the impaired SCCD group, insufficient sTLR4 compensation likely led to a diminished capacity to neutralize HMGB1/IL-1β, resulting in sustained inflammatory signaling, potentially amplified by oxidative stress, and ultimately compromised sperm chromatin structure (Chu et al., 2018). This finding provides a valuable perspective on sperm quality control under inflammatory conditions and highlights the importance of both the sTLR4/HMGB1 and sTLR4/IL-1β ratios as potential biomarkers for assessing immune homeostasis in the male reproductive tract.

Correlation analysis revealed that seminal pro-inflammatory factors, like HMGB1, IL-1β, and TNF-α, were significantly positively correlated with each other. These factors were also intertwined with oxidative stress markers, forming a synergistic “inflammatory-oxidative” network that may drive sperm chromatin damage. Within this context, the sTLR4/HMGB1 and sTLR4/IL-1βratios exhibited robust negative correlations with the SCCD rate, with association strengths far exceeding that of sTLR4 alone. This finding suggests that the sTLR4 system acts not as a passive inflammatory byproduct but as a critical “immunological buffer”: higher ratios indicate a greater capacity of sTLR4 to competitively bind ligands like HMGB1, blocking their interaction with mTLR4 and thereby inhibiting the overactivation of the NF-κB pathway to protect sperm nuclear assembly from inflammatory damage at the source (Prescott et al., 2021). Consequently, these two ratios provide a more accurate reflection of immune homeostasis in the reproductive tract than any single metric.

The aforementioned correlation analysis uncovered a complex covariation network among biomarkers and preliminarily highlighted the prominent negative associations of the sTLR4/HMGB1 and sTLR4/IL-1β ratios with SCCD incidence. To explore the independence of these associations, we employed a Firth penalized logistic regression model. Given the modest sample size, the estimate for T-AOC exhibited an extreme odds ratio with a very wide confidence interval, consistent with complete or quasi-complete separation, a known artifact in small-sample logistic modeling that Firth regression attenuates but does not fully eliminate (Heinze and Schemper, 2002). In contrast, the sTLR4/IL-1β ratio, despite showing an odds ratio approaching zero, had a relatively narrow confidence interval and remained statistically significant after multivariable adjustment (**Table 4**), suggesting that its association with SCCD is more robust. Therefore, the univariate T-AOC result should be interpreted with caution and requires validation in larger cohorts.

Despite this estimation uncertainty, the sTLR4/IL-1β ratio remained associated with reduced SCCD risk, consistent with its role in regulating the seminal inflammatory microenvironment. IL-1β directly impairs sperm DNA and triggers apoptosis via caspase activation and mitochondrial ROS generation (Paira et al., 2022), while sTLR4 counteracts these effects by acting as a decoy receptor that competitively binds DAMPs and negatively regulates TLR4/NF-κB signaling (Mansouri et al., 2025). Thus, a higher sTLR4/IL-1β ratio likely reflects preserved molecular buffering against inflammatory pressure. Notably, the sTLR4/HMGB1 ratio lost significance in the regression model despite a negative bivariate correlation, suggesting its effect may be mediated through the IL-1β pathway or attenuated by sample variability.

Although univariate Firth regression identified elevated T-AOC as significantly associated with increased SCCD risk, this association was attenuated and no longer statistically significant after adjusting for age, abstinence duration, semen pH, and total sperm motility (*P* = 0.472). This attenuation is biologically plausible and likely reflects confounding by clinical and biochemical covariates rather than a true independent effect of T-AOC. Seminal plasma TAC is not a static trait but an integrative readout of enzymatic and non-enzymatic antioxidants whose levels are modulated by age, hormonal status, abstinence period, and the degree of local inflammation (Mancini et al., 2009). In our cohort, T-AOC was negatively correlated with IL-1β (r = -0.25, *P* < 0.05) and tended to track with a more alkaline seminal pH in the impaired SCCD group, suggesting that higher T-AOC may partly represent a compensatory upregulation of antioxidant defenses against preexisting oxidative insult rather than a primary protective factor (Subramanian et al., 2018; Gupta et al., 2021). This pattern, where antioxidant capacity appears elevated under oxidative pressure, has been described previously: under sustained inflammatory or oxidative pressure, spermatozoa and accessory glands may increase antioxidant output, producing a positive correlation between T-AOC and the severity of chromatin damage (Agarwal et al., 2005). Furthermore, because T-AOC aggregates the net reductive capacity without specifying which antioxidants are responsible, its crude value can be confounded by lifestyle, glandular secretion shifts, and pH-dependent antioxidant stability (Mahfouz et al., 2009). Given that T-AOC lost significance after multivariable adjustment, the univariate finding should be interpreted with caution and regarded as an exploratory observation rather than evidence of an independent effect. Future studies employing targeted assays for individual antioxidants are warranted to clarify the underlying mechanisms.

In contrast to the inconsistent findings for T-AOC, the sTLR4/IL-1β ratio demonstrated a more robust and clinically meaningful discriminatory performance.​The ROC curve analysis demonstrated that the sTLR4/IL-1β ratio achieved an AUC of 0.786 for discriminating SCCD from controls, indicating moderate-to-good discriminatory ability that is consistent with its independent association with reduced SCCD risk in the multivariable Firth regression (*P* < 0.001). This AUC is in line with the diagnostic performance reported for other seminal inflammatory biomarkers in male infertility research. For instance, IL-1β and IL-6 in seminal plasma have been shown to differentiate infertile from fertile men and to correlate with sperm motility loss, with ROC accuracies for IL-1β and oxidative stress markers reaching AUC ≈ 0.74 in related studies (Omar and Berwary, 2025). Similarly, IL-6 and TNF-α elevations in seminal plasma are well documented in infectious/inflammatory subfertility, although these markers are inherently confounded by leukocytospermia and BMI (Eggert-Kruse et al., 2007). Thus, the sTLR4/IL-1β ratio offers an advantage by capturing the dynamic balance between decoy-receptor buffering and proximal cytokine amplification. It should be noted that the AUC of 0.786 was derived from a relatively small cohort without internal validation and therefore may be somewhat optimistic; validation in independent external cohorts is needed before clinical application.​ Nevertheless, an AUC of 0.786 approaches the 0.80 threshold commonly regarded as indicative of good discriminatory ability for a screening tool (Mandrekar, 2010). This is not surprising given that SCCD is multifactorial and also shaped by oxidative stress, hormonal, and genetic contributions beyond inflammation alone. Future models incorporating oxidative stress markers or sperm DNA fragmentation indices may further enhance discrimination. Overall, the sTLR4/IL-1β ratio represents a valuable non-invasive indicator whose utility is reinforced by its biological plausibility and its consistent signal across correlation, multivariable regression, and ROC analyses in this cohort.

In the present study, several biomarkers exhibited extreme odds ratios, such as, T-AOC: OR = 200.99, sTLR4/IL-1β: OR ≈ 0.00, with exceptionally wide confidence intervals. While Firth penalized regression was employed to reduce bias from small-sample separation, these estimates still reflect considerable instability. Importantly, these extreme values do not necessarily imply strong biological effects; rather, they signal that the magnitude of association remains poorly defined. Therefore, we emphasize that the primary value of these findings lies in the directionality of association rather than the absolute effect size. Future studies with larger cohorts and balanced exposure distributions are warranted to validate and refine these estimates.

## Conclusions and limitations

In conclusion, our study identifies the sTLR4/IL-1β ratio as a robust independent biomarker associated with reduced SCCD risk, underscoring the importance of inflammatory homeostasis in male fertility. The attenuation of T-AOC significance after multivariable adjustment highlights the confounding effects of clinical covariates and the need for cautious interpretation of univariate antioxidant signals. The sTLR4/IL-1β ratio demonstrated moderate-to-good discriminatory ability. However, its clinical utility requires validation in larger, independent cohorts to confirm the effect size and improve estimation precision. Therefore, the present findings should be considered exploratory and hypothesis-generating.

Several limitations should be acknowledged. First, the cross-sectional design precludes causal inference. Second, the modest sample size may limit generalizability and the discriminative performance of the sTLR4/IL-1β ratio requires validation in larger, independent cohorts. Third, the number of biomarker comparisons increases the risk of false-positive findings; therefore, the observed associations require independent replication before definitive conclusions can be drawn. Fourth, unmeasured confounders, such as lifestyle factors, genetic variations, may have influenced the results. Future studies may benefit from analyzing SCCD as a continuous variable and adopting a narrower abstinence window to minimize potential confounding. Finally, our study stratified participants based on SCCD rate rather than clinical fertility status; the inclusion of a fertile control group in future studies would help validate the clinical relevance of these findings.

Despite these limitations, this study provides a foundation for future research. Prospective studies with larger, multicenter cohorts are needed to validate the sTLR4/IL-1βratio as a clinical tool for SCCD risk assessment and to explore therapeutic strategies targeting the sTLR4-mediated inflammatory buffering system.

## Data Availability

The raw data supporting the conclusions of this article will be made available by the authors, without undue reservation.

## References

[B1] Agarwal A. Gupta S. Sharma R. K. (2005). Role of oxidative stress in female reproduction. Reprod. Biol. Endocrinol. 3, 28. doi: 10.1186/1477-7827-3-28 16018814 PMC1215514

[B2] Cao H. Liu S. Cui S. Nie H. Liu X. Qin W. (2025). From stress signals to fertility challenges: The role of damage-associated molecular patterns in male reproduction. Front. Immunol. 16, 1598451. doi: 10.3389/fimmu.2025.1598451 41103424 PMC12521105

[B3] Chu M. Zhou M. Jiang C. Chen X. Guo L. Zhang M. . (2018). Staphylococcus aureus phenol-soluble modulins α1-α3 act as novel toll-like receptor (TLR) 4 antagonists to inhibit HMGB1/TLR4/NF-κB signaling pathway. Front. Immunol. 9, 862. doi: 10.3389/fimmu.2018.00862 29922279 PMC5996891

[B4] Dinarello C. A. (2009). Immunological and inflammatory functions of the interleukin-1 family. Annu. Rev. Immunol. 27, 519–550. doi: 10.1146/annurev.immunol.021908.132612 19302047

[B5] Eggert-Kruse W. Kiefer I. Beck C. Demirakca T. Strowitzki T. (2007). Role for tumor necrosis factor alpha (TNF-alpha) and interleukin 1-beta (IL-1beta) determination in seminal plasma during infertility investigation. Fertil. Steril 87, 810–823. doi: 10.1016/j.fertnstert.2006.08.103 17430733

[B6] Fraga C. G. Oteiza P. I. Galleano M. (2014). *In vitro* measurements and interpretation of total antioxidant capacity. Biochim. Biophys. Acta 1840, 931–934. doi: 10.1016/j.bbagen.2013.06.030 23830861

[B7] Gao J. Yuan R. Yang S. Wang Y. Huang Y. Yan L. . (2021). Age-related changes in human conventional semen parameters and sperm chromatin structure assay-defined sperm DNA/chromatin integrity. Reprod. Biomed. Online 42, 973–982. doi: 10.1016/j.rbmo.2021.02.006 33785305

[B8] Garcia-Segura S. Ribas-Maynou J. Lara-Cerrillo S. Garcia-Peiró A. Castel A. B. Benet J. . (2020). Relationship of seminal oxidation-reduction potential with sperm DNA integrity and pH in idiopathic infertile patients. Biol. (Basel) 9, 262. doi: 10.3390/biology9090262 32882928 PMC7564726

[B9] Gupta S. Finelli R. Agarwal A. Henkel R. (2021). Total antioxidant capacity-relevance, methods and clinical implications. Andrologia 53, e13624. doi: 10.1111/and.13624 32400041

[B10] Hammadeh M. E. Zeginiadov T. Rosenbaum P. Georg T. Schmidt W. Strehler E. (2001). Predictive value of sperm chromatin condensation (aniline blue staining) in the assessment of male fertility. Arch. Androl 46, 99–104. doi: 10.1080/01485010117363 11297072

[B11] Heinze G. Schemper M. (2002). A solution to the problem of separation in logistic regression. Stat. Med. 21, 2409–2419. doi: 10.1002/sim.1047 12210625

[B12] Jomova K. Raptova R. Alomar S. Y. Alwasel S. H. Nepovimova E. Kuca K. . (2023). Reactive oxygen species, toxicity, oxidative stress, and antioxidants: chronic diseases and aging. Arch. Toxicol. 97, 2499–2574. doi: 10.1007/s00204-023-03562-9 37597078 PMC10475008

[B13] Liu K. Chen Y. An R. (2024). The mechanism and clinical significance of sperm DNA damage in assisted reproductive. Front. Biosci. (Landmark Ed) 29, 416. doi: 10.31083/j.fbl2912416 39735980

[B14] Mahfouz R. Sharma R. Sharma D. Sabanegh E. Agarwal A. (2009). Diagnostic value of the total antioxidant capacity (TAC) in human seminal plasma. Fertil. Steril 91, 805–811. doi: 10.1016/j.fertnstert.2008.01.022 18314108

[B15] Mancini A. Festa R. Silvestrini A. Nicolotti N. Di Donna V. La Torre G. . (2009). Hormonal regulation of total antioxidant capacity in seminal plasma. J. Androl 30, 534–540. doi: 10.2164/jandrol.108.006148 19234315

[B16] Mandrekar J. N. (2010). Receiver operating characteristic curve in diagnostic test assessment. J. Thorac. Oncol. 5, 1315–1316. doi: 10.1097/JTO.0b013e3181ec173d 20736804

[B17] Mansouri A. Akthar I. Miyamoto A. (2025). TLR2 and TLR4 bridge physiological and pathological inflammation in the reproductive system. Commun. Biol. 8, 1008. doi: 10.1038/s42003-025-08424-x 40618011 PMC12228841

[B18] Naseer Z. Mudussar N. Ahmad E. Zia ur R. (2024). Oxidative stress and male fertility: Promising role of nutraceuticals. Biochem. IntechOpen. doi: 10.5772/intechopen.112304

[B19] Omar D. A. Berwary N. J. A. (2025). Immunomodulation, hormonal imbalance related to bisphenol A induced male infertility. Karbala J. Med. 18, 3045–3054. doi: 10.70863/karbalajm.v18i2.4941

[B20] Oumaima A. Tesnim A. Zohra H. Amira S. Ines Z. Sana C. . (2018). Investigation on the origin of sperm morphological defects: oxidative attacks, chromatin immaturity, and DNA fragmentation. Environ. Sci. pollut. Res. Int. 25, 13775–13786. doi: 10.1007/s11356-018-1417-4 29508198

[B21] Paira D. A. Silvera-Ruiz S. Tissera A. Molina R. I. Olmedo J. J. Rivero V. E. . (2022). Interferon γ, IL-17, and IL-1β impair sperm motility and viability and induce sperm apoptosis. Cytokine 152, 155834. doi: 10.1016/j.cyto.2022.155834 35217429

[B22] Pérez-Soto E. Oros-Pantoja R. Fernández-Martínez E. Carbonell-Campos J. M. Sánchez Monroy V. (2021). Seminal pro-inflammatory cytokines and pH are affected by Chlamydia infection in asymptomatic patients with teratozoospermia. Cent. Eur. J. Immunol. 46, 76–81. doi: 10.5114/ceji.2021.105247 33897287 PMC8056351

[B23] Petelin A. Tedeschi P. Maietti A. Jurdana M. Brandolini V. Pražnikar Z. J. (2017). Total serum antioxidant capacity in healthy normal weight and asymptomatic overweight adults. Exp. Clin. Endocrinol. Diabetes 125, 470–477. doi: 10.1055/s-0043-107783 28445896

[B24] Prescott J. A. Mitchell J. P. Cook S. J. (2021). Inhibitory feedback control of NF-κB signalling in health and disease. Biochem. J. 478, 2619–2664. doi: 10.1042/BCJ20210139 34269817 PMC8286839

[B25] Sellami A. Chakroun N. Ben Zarrouk S. Sellami H. Kebaili S. Rebai T. . (2013). Assessment of chromatin maturity in human spermatozoa: useful aniline blue assay for routine diagnosis of male infertility. Adv. Urol. 2013, 578631. doi: 10.1155/2013/578631 24198830 PMC3808709

[B26] Subramanian V. Ravichandran A. Thiagarajan N. Govindarajan M. Dhandayuthapani S. Suresh S. (2018). Seminal reactive oxygen species and total antioxidant capacity: Correlations with sperm parameters and impact on male infertility. Clin. Exp. Reprod. Med. 45, 88–93. doi: 10.5653/cerm.2018.45.2.88 29984209 PMC6030612

[B27] World Health Organization (2021). WHO laboratory manual for the examination and processing of human semen. 6th ed (Geneva: World Health Organization). Available online at: https://iris.who.int/handle/10665/343208 (Accessed Aug 14, 2026).

[B28] Zhang Y. Karki R. Igwe O. J. (2015). Toll-like receptor 4 signaling: A common pathway for interactions between prooxidants and extracellular disulfide high mobility group box 1 (HMGB1) protein-coupled activation. Biochem. Pharmacol. 98, 132–143. doi: 10.1016/j.bcp.2015.08.109 26367307 PMC4600681

[B29] Zhou W. Lu S. Wang A. Jiang M. Li S. Zhou H. (2026). Sperm chromatin condensation defects and IVF outcomes: A retrospective cohort study. PeerJ 14, e20749. doi: 10.7717/peerj.20749 41630849 PMC12861134

